# Spatio-temporal dynamics of EEG features during sleep in major depressive disorder after treatment with escitalopram: a pilot study

**DOI:** 10.1186/s12888-020-02519-x

**Published:** 2020-03-14

**Authors:** Li Wu, Xue-Qin Wang, Yong Yang, Teng-Fei Dong, Ling Lei, Qi-Qi Cheng, Su-Xia Li

**Affiliations:** 1 School of automation Hangzhou Dianzi University, HangZhou Economic Development Zone, 1158, 2# Road, BaiYang Street, Hangzhou, 310018 Zhejiang China; 2 National Institute on Drug Dependence, Peking University, Beijing, 100191 China; 3 Peking University Sixth Hospital, Peking University Institute of Mental Health, NHC Key Laboratory of Mental Health (Peking University), National Clinical Research Center for Mental Disorders (Peking University Sixth Hospital), Beijing, 100191 China

**Keywords:** EEG, Major depressive disorder, Escitalopram, Power spectrum, Nonlinear dynamics, Spatio- temporal dynamics

## Abstract

**Background:**

Previous studies have shown escitalopram is related to sleep quality. However, effects of escitalopram on dynamics of electroencephalogram (EEG) features especially during different sleep stages have not been reported. This study may help to reveal pharmacological mechanism underlying escitalopram treatment.

**Methods:**

The spatial and temporal responses of patients with major depressive disorder (MDD) to escitalopram treatment were analyzed in this study. Eleven MDD patients and eleven healthy control subjects who completed eight weeks’ treatment of escitalopram were included in the final statistics. Six-channel sleep EEG signals were acquired during sleep. Power spectrum and nonlinear dynamics were used to analyze the spatio-temporal dynamics features of the sleep EEG after escitalopram treatment.

**Results:**

For temporal dynamics: after treatment, there was a significant increase in the relative energy (RE) of δ_1_ band (0.5 - 2 Hz), accompanied by a significant decrease in the RE of β_2_ band (20 - 30 Hz). Lempel-Ziv complexity and Co - complexity values were significantly lower. EEG changes at different sleep stages also showed the same regulation as throughout the night sleep. For spatio dynamics: after treatment, the EEG response of the left and right hemisphere showed asymmetry. Regarding band-specific EEG complexity estimations, δ1 and β2 in stage-1 and δ1 in stage-2 sleep stage in frontal cortex is found to be much more sensitive to escitalopram treatment in comparison to central and occipital cortices.

**Conclusions:**

The sleep quality of MDD patients improved, EEG response occurred asymmetry in left and right hemispheres due to escitalopram treatment, and frontal cortex is found to be much more sensitive to escitalopram treatment. These findings may contribute to a comprehensive understanding of the pharmacological mechanism of escitalopram in the treatment of depression.

## Background

Major depressive disorder (MDD) is one of the most common diseases worldwide and is responsible for premature deaths and disability [7]. In the past, MDD patients have been treated mainly with traditional drugs such as monoamine oxidase inhibitors, or tricyclic and heterocyclic antidepressants [20, 43]. These traditional drugs have some disadvantages such as weak tolerance, large adverse effects and slow action onset. Escitalopram is a highly selective SSRI and therapeutically active S-enantiomer of citalopram, it has been widely used and recommended by clinicians worldwide [5]. Compared to other antidepressants, escitalopram is tolerated better, has fewer adverse effects and has a faster onset of action [5, 27, 36, 39]. However, its pharmacological mechanism has not yet been clarified completely.

Previous study has shown escitalopram can improve sleep quality in MDD patients [29], and was efficacious in treating depressive symptoms in depressed patients suffering from poor sleep quality, and this beneficial effect appeared to be independent of the severity of the patient’s sleep problems [30]. Furthermore, it has been reported that escitalopram is advantageous in the treatment of the core symptoms of MDD, including sleep disturbance [42]. Sleep is a complicated process, and can be divided into different stages both temporally, and spatially, and is related to multiple interactions between different brain regions. Therefore, it is necessary to explore the pharmacological mechanism underlying the action of escitalopram, and to investigate the relationship between escitalopram and sleep in both temporal and spatial dimensions. However, until now, there have been few studies involving both temporal and spatial dimensions, only research involving the eye close and open condition [8]. Currently there are questions still to be resolved, such as whether escitalopram acts the same at each sleep stage, or is specific to certain stages. Also it remains unclear as to whether escitalopram is brain region specific such as the frontal, central, or occipital cortices or displays asymmetries in brain hemispheres.

Electroencephalography (EEG) is a suitable option as a neurophysiological biomarker, and displays several advantages, including higher temporal resolution, non-invasiveness, ease of access and low cost [15, 33]. As a result this method has been widely used for the biomedical investigation of several mental illnesses including MDD, Alzheimer’s disease and others [1, 2, 6, 22]. EEG signals can be analyzed by linear and non-linear dynamic analyses. Linear analysis such as power spectrum is commonly used to extract the features of sleep EEG signals [13, 26, 40, 44]. Spectral characteristic parameters can reflect the energy information transported by each frequency band. Because the brain is a complex non-linear system, the use of non-linear dynamic analysis may also be used to reflect brain states accurately [24, 25, 35]. Among the non-linear features, complexity is suitable as it can be calculated within a short time series and fast speed.

In the present study, we investigated the spatio-temporal dynamics of sleep EEG features before and after escitalopram treatment. Both linear and non-linear dynamic analyses may provide a more comprehensive understanding of the pharmacological mechanism of escitalopram in the treatment of depression.

## Methods

### Participants

A total of 58 subjects participated in the study, which included 30 MDD patients and 28 healthy controls. However, more than half of MDD patients were excluded from the final analysis because their electrode became detached, more than half of healthy controls were eliminated because their sleep time was less than 6.5 h on the experimental night. Finally, 11 healthy male adult controls and 11 male MDD patients completed the study. The MDD patients ages’ ranged from 22 to 40 years (mean ± SD: 30.64 ± 5.52 years). These patients reported no history of any other psychiatric disorder or prior take of antidepressants. All patients, were from The Peking University Sixth Hospital, and met the criteria for major depression defined in the Diagnostic and Statistical Manual of Mental Disorders, 5th edition (DSM-5) [3]. Diagnosis was established by experienced psychiatrists using the Structured Clinical Interview for DSM-5: Research Version (SCID) [18]. A minimum score of 22 points on the 17 item Hamilton Depression Scale (HRSD - 17) [41] was required to be enrolled in the study. Professional scorers from the Peking University Sixth Hospital conducted the HRSD measurements two times. The first time occurred before the treatment of escitalopram, and the second time occurred on day 57 of escitalopram treatment. The exclusion criteria included: (1) age < 18 or 45 > years, (2) presence of additional psychotic symptoms, (3) cognitive impairment or personality disorders, (4) history of other mental illness, (5) suicidal ideation or behaviors.

Control participants included 11 physically and mentally healthy male volunteers whose ages were between 22 and 38 years (mean ± SD: 27.72 ± 4.79 years). The inclusion criteria included: (1) self-reported good sleep and PSQI < 5, matched age with MDD, (2) absence of psychiatric illnesses diagnosed by the DSM-5 criteria, (3) a maximum score of 7 points on the 17 - item HRSD, (4) a maximum score of 7 points on the 14 - item Hamilton Anxiety Scale (HAMA) [34], (5) 18 ≤ BMI < 30.

The exclusion criteria included: (1) any of the exclusion criteria for the MDD group, (2) any past or present history of mental illness that met DSM-5 diagnostic criteria, (3) current or pass chronic physical diseases (e.g., cardiovascular disease, diabetes, rheumatoid arthritis, et al.), (4) shift worker within the preceding year, (5) jet lag travel in the last 2 weeks, (5) total sleep time < 6.5 h.

All of the participants were Han Chinese. They signed written informed consent forms before participation. The study was approved by the ethics committee of Peking University Sixth Hospital, Beijing, China, in accordance with the Helsinki Declaration.

### Polysomnographic recording

All the depressive patients underwent polysomnographic recording two times. The first time conducted before the treatment of escitalopram, and the second time conducted on day 57 of escitalopram treatment.

Overnight polysomnographic recording included electroencephalography (EEG; including F3, F4, C3, C4, O1, and O2, with reference to the contralateral mastoid; International10 - 20system), electrooculography (EOG), electromyography (EMG), and electrocardiography (ECG). The signals were digitized at a sampling rate of 256 Hz, and an electrode impedance < 5 KΩ. Thirty - second epochs were used for manual analysis, and sleep stages were scored offline according to the criterion of the American Academy of Sleep Medicine (AASM) [9], using the standard polysomnographic sleep recordings.

### EEG signal processing

In the processing environment of MATLAB R2016b, using EEGLAB toolkits (University of California San Diego), power frequency interference was eliminated by using a 50 Hz notch, and data was filtered from 0.5 to 30 Hz by using band pass filter [16]. Each sample had corresponding sleep staging files. However, because the sample duration data was too large, and some data frames had large artifacts, we chose the entire artifact - free frames (30 s) from every sleep type (including Wake, rapid-eye-movement (REM), stage-1, stage-2 (including sleep spindles) and stage-3) according to sleep staging files.

The EEG signal processing includes two aspects: linear analysis and nonlinear dynamic analysis.

#### Linear analysis: power spectrum

The power spectrum reflects the energy information carried by the brain waves in each frequency band. According to the frequency, the EEG signals are divided into several categories: δ_1_ (0.5 - 2 Hz), δ_2_ (2 - 4 Hz), θ (4 - 8 Hz), α (8 - 13 Hz), β_1_ (13 - 20 Hz) and β_2_ (20 - 30 Hz) [14]. A previous study revealed that during the night, the frequencies of the most powerful waves are concentrated in the 0.5–2 Hz range and show a continuous tendency to shift towards slower frequencies during sleep. So we divided the delta band into low-delta (0.5–2 Hz) and high-delta (2 - 4 Hz )[31]. In the present study, each frequency band power was obtained by using fast Fourier transform (FFT) analysis [17, 38, 45]. FFT calculation was performed on 3 s non-overlapping consecutive window (Hamming window). The average values of the different sleep stages were computed form the 30 s of data obtained previously. In order to reduce specific individual differences, the relative energy (RE) was computed. The RE corresponds to the ratio between the power value of each frequency band and the sum of the power values in the following calculation:

$$ \boldsymbol{Relative}\ \boldsymbol{energy}=\frac{\boldsymbol{Power}\ \boldsymbol{value}\ \boldsymbol{of}\ \boldsymbol{a}\ \boldsymbol{s}\boldsymbol{pecific}\ \boldsymbol{band}}{\boldsymbol{Sum}\ \boldsymbol{of}\ \boldsymbol{the}\ \boldsymbol{power}\ \boldsymbol{value}\boldsymbol{s}}\ \left(\mathbf{1}\right) $$

#### Nonlinear dynamic analysis: LZC, C0C

Correlation dimension, complexity, entropy and Lyapunov exponents are common non-linear features in EEG signal analysis. Correlation dimension and Lyapunov exponents require large data sets and strict dimensional measurements which are not suitable for EEG analysis. Whereas Lempel-Ziv Complexity (LZC) and Co-complexity (C0C) are more suitable, because they require small datasets and have high computation speeds. Therefore, in the present study, LZC and C0C were used to characterize the sleep state of patients with MDD. LZC represents the rate of appearance of a new pattern in a time series from a one dimensional perspective [32]. A ratio of the area of the disorder component over the area of the original time series is considers as a complexity measurement, which is denoted as C 0[12]. The higher the LZC, the more likely it is that a new model will appear, highlighting complex dynamic behavior. The higher the C0C, the more probability there is that random motion may appear.

In order to obtain 28 s sequences, the first and last second of each of them were removed from the 30 s previously selected sequences. This shortened sequence was then divided into 7 segments of 4 s each for targeted analysis. For each of those time-windows we considered as important EEG features, the mean of the values by itself as well as the characteristic of values according to the sleep stage.

### Statistical analysis

All analyses were performed using the SPSS Statistics version 22.0. We used the paired-samples t-test to investigate the changes in the EEG characteristic parameters between the baseline (before treatment) and the final (after treatment) session, and the independent-sample t-test to compare the results from the patients with MDD and healthy control subjects. We then used the paired-samples t-test to analyze differences between the left and right hemispheres of the cortex. The differences of EEG characteristics in different brain regions (frontal, central, occipital) between baseline and final were analyzed by one-way ANOVA. Differences were considered significant when *P* < 0.05.

## Results

### EEG response before and after escitalopram treatment of the whole night sleep

**Fig. 1:**
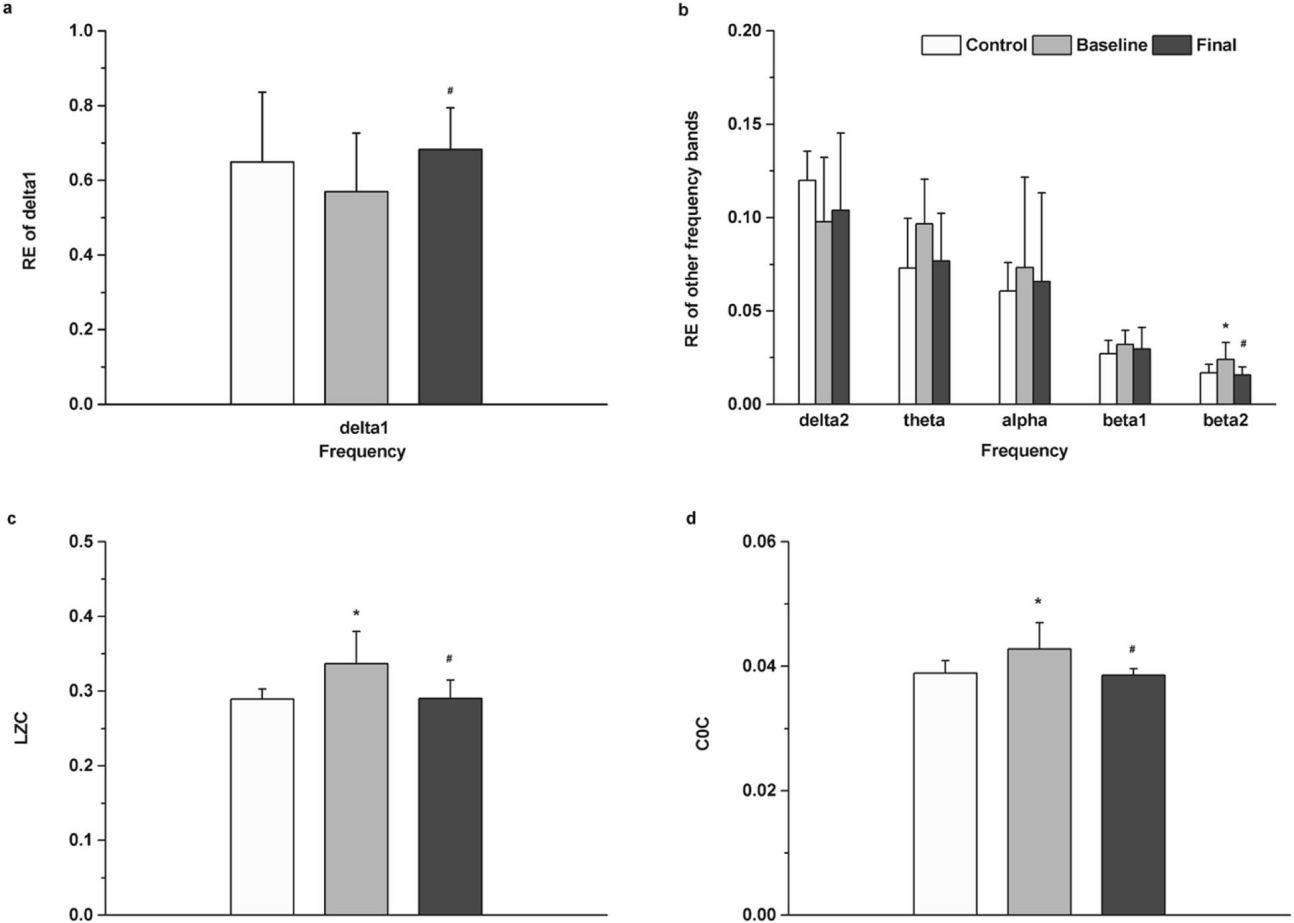
EEG response before and after escitalopram treatment during the whole night sleep. **a** Relative energy (RE) in the δ_1_ band; **b** RE of other frequency bands (except delta1); **c** LZC values; **d** C0C values; The data are expressed as mean ± SD. *n* = 11 MDD patients. n = 11 healthy controls. *p < 0.05, different from control, ^#^*p* < 0.05, different from baseline

We investigated the EEG response for MDD patients (before and after escitalopram treatment) and healthy control subjects in the spatial and temporal dimensions. For temporal dynamic analysis, firstly, we compared the RE (average value of six channels) of different EEG frequency bands of the whole night sleep between MDD patients (before and after escitalopram treatment) and healthy control subjects. Figure 1 (a & b) indicate that after treatment, the RE of the δ1 band was significantly higher than that before treatment (*t*_(10)_ = − 2.397, *p* = 0.028). The RE of the β2 band in patients with MDD before treatment with escitalopram was significantly higher than that in controls (*t*_(20)_ = 2.513, *p* = 0.045), it was significantly decreased to control level after treatment (*t*_(10)_ = 2.513, *p* = 0.045). Additionally, the RE of other frequency bands in MDD patients also had some improvement after treatment, but not statistically significant.

Secondly, we analyzed whether there existed differences in non-linear LZC and C0C values (average value of six channels) during the whole night sleep for MDD patients (before and after escitalopram treatment) and healthy control subjects. As detailed in Fig. 1 (c & d), the LZC values in patients before treatment with escitalopram was higher than that in controls (*t*_(20)_ = 2.963, *p* = 0.010), it was significantly decreased to control level after treatment (*t*_(10)_ = 2.626, *p* = 0.030). Furthermore, the C0C values showed the same regulation as LZC values, the C0C values in patients before treatment with escitalopram was higher than that in controls (*t*_(20)_ = 2.397, *p* = 0.028), it was significantly decreased to control level after treatment (*t*_(10)_ = 2.862, *p* = 0.007).

### EEG response before and after escitalopram treatment at different sleep stages

**Fig. 2:**
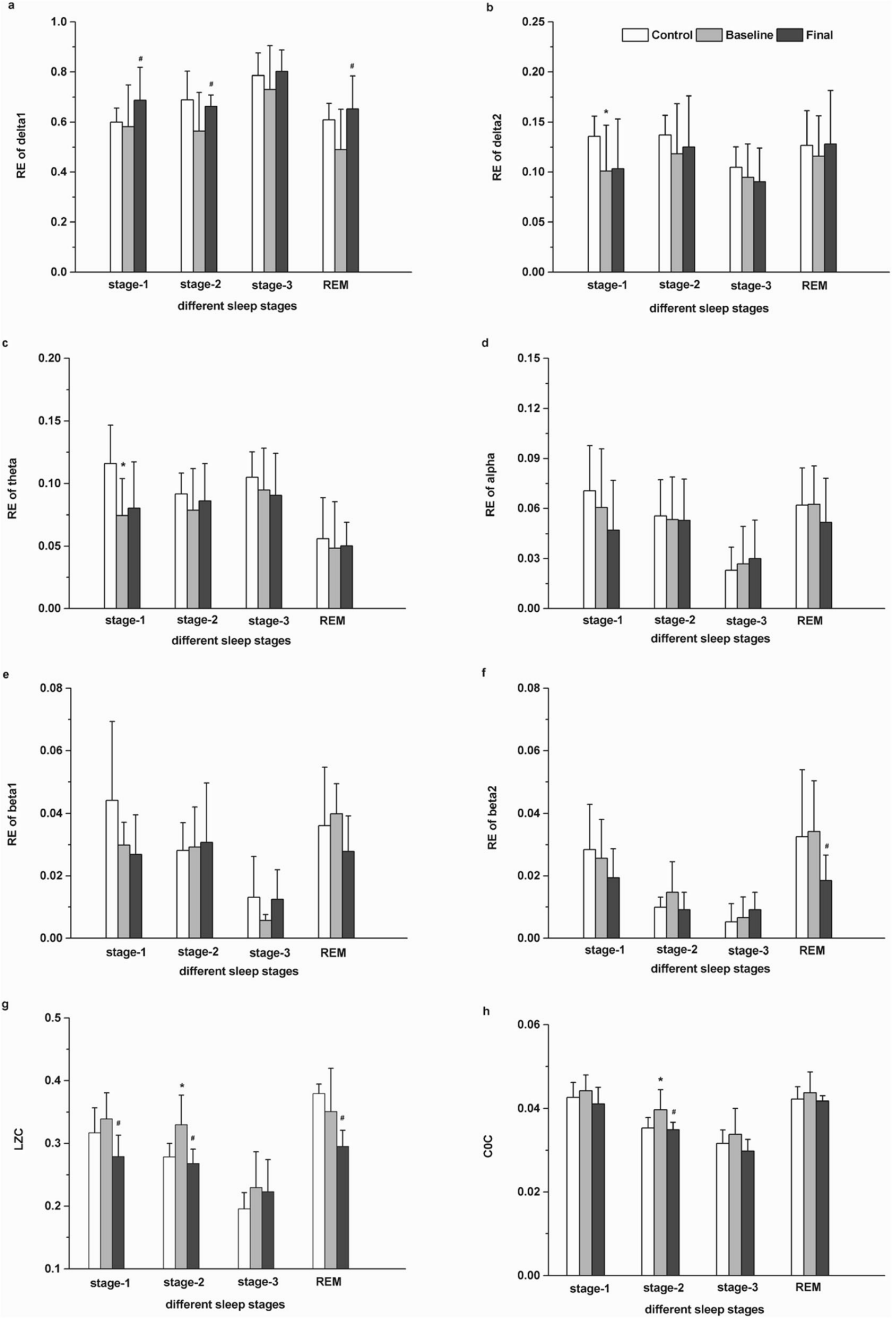
EEG response before and after escitalopram treatment during each sleep stage. **a** Relative energy (RE) in the δ_1_ band; **b** RE in the δ_2_ band; **c** RE in the θ band; **d** RE in the α band; **e** RE in the β_1_ band; **f** RE in the β_2_ band; **g** LZC values; **h** C0C values; The data are expressed as mean ± SD. n = 11 MDD patients. n = 11 healthy controls. *p < 0.05, different from control, ^#^*p* < 0.05, different from baseline

We compared the changes in RE (average value of six channels) of each frequency bands at different sleep stages in MDD patients (before and after escitalopram treatment) and healthy controls. Firstly, we compared the RE of each frequency bands during different sleep stages. As shown in Fig. 2a, a significant increase in the RE of δ_1_ band in the stage-1 (*t*_(10)_ = − 2.239, *p* = 0.049), stage-2 (*t*_(10)_ = − 2.923, *p* = 0.015), and REM (*t*_(10)_ = − 2.648, *p* = 0.024) sleep stages after treatment with escitalopram was seen. Additionally, a significant decrease in the RE of δ_2_ band (*t*_(20)_ = − 2.371, *p* = 0.027), and θ band (*t*_(20)_ = − 2.923, *p* = 0.004) in the stage-1 in patients with MDD before treatment compared with controls (Fig. 2b and c). A significant decrease of the β_2_ band in the REM (*t*_(10)_ = 3.126, *p* = 0.011) sleep phase in patients with MDD after treatment compared with before treatment (Fig. 2f).

Secondly, we analyzed for differences in non-linear LZC and C0C values (average value of six channels) at different sleep stages in MDD patients and the healthy control subjects. As shown in Fig. 2g, LZC values in patients with MDD after treatment decreased significantly compared with that before treatment during stage-1 (*t*_(10)_ = 3.946, *p* = 0.003), stage-2 (*t*_(10)_ = 3.527, *p* = 0.005) and REM (*t*_(10)_ = 2.920, *p* = 0.015) sleep stage. While during stage-2 LZC values in patients with MDD before treatment increased significantly compared with that in controls (t _(20)_ = 2.847, *p* = 0.011). As for the C0C values, during stage-2 showed a significant increase in patients with MDD before treatment compared with that in controls (*t*_(10)_ = 2.387, *p* = 0.029) and a significantly decreased in patients with MDD after treatment compared with that before treatment (*t*_(10)_ = 3.126, *p* = 0.011) in patients with MDD (Fig. 2h).

### EEG response before and after escitalopram treatment in left and right hemispheres

**Fig. 3:**
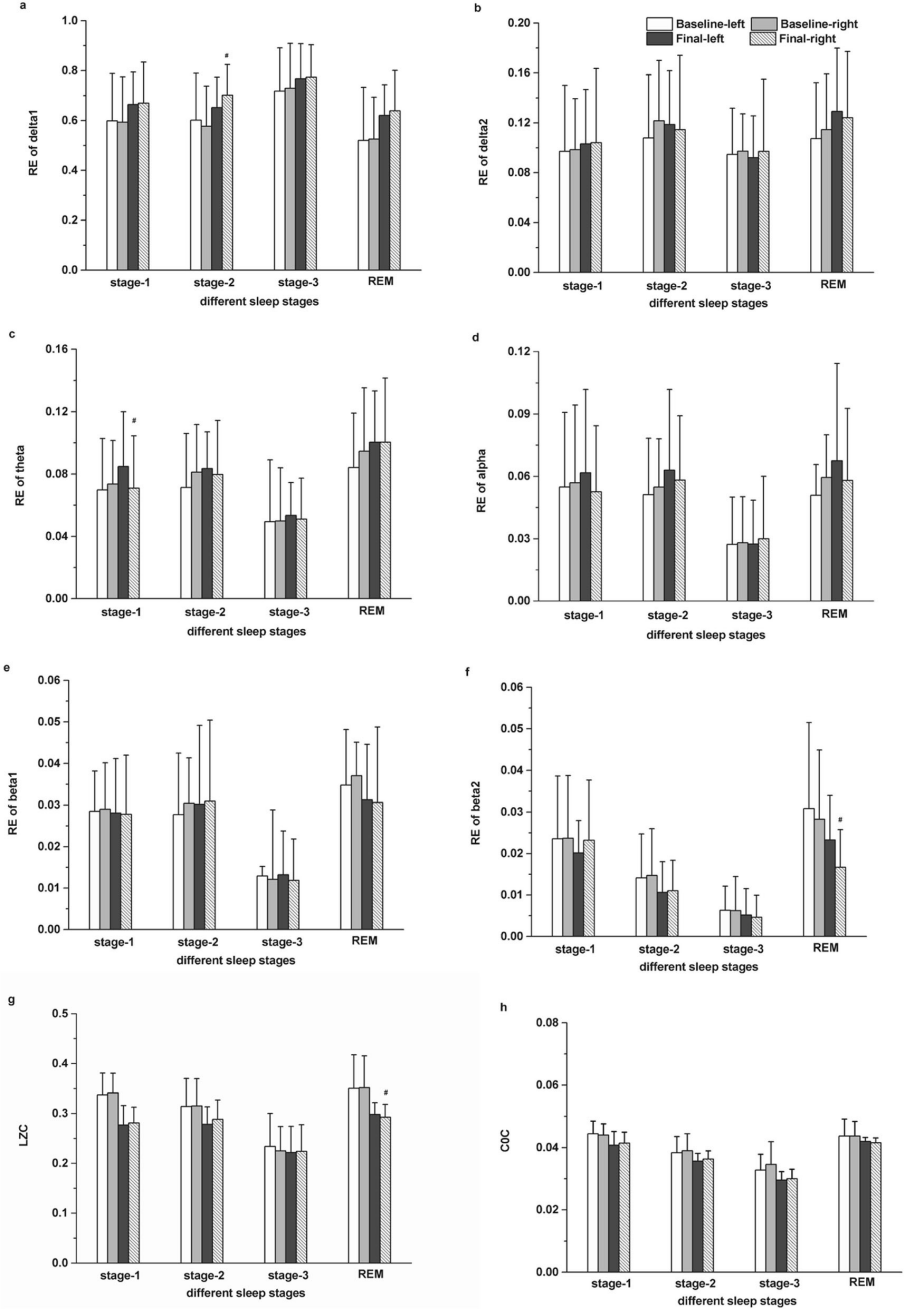
EEG response before and after escitalopram treatment in left and right hemispheres at different sleep stages. **a** Relative energy (RE) in the δ_1_ band; **b** RE in the δ_2_ band; **c** RE in the θ band; **d** RE in the α band; **e** RE in the β_1_ band; **f** RE in the β_2_ band; **g** LZC values; **h** C0C values. The data are expressed as mean ± SD. n = 11 MDD patients. n = 11 healthy controls. ^#^*p* < 0.05, different from final - left

For spatio dynamic analysis, we found differences in EEG features between left and right hemispheres in MDD patients (before and after escitalopram treatment). EEG features of left hemisphere were average values of three channels including F3, C3, and O1; EEG features of right hemisphere were average values of another three channels including F4, C4, and O2. Firstly, we analyzed for differences in the RE between left and right hemispheres during different sleep stages. As shown in Fig. 3a, the RE of the δ_1_ band during stage-2 in the right hemisphere greater significantly compared with that in the left hemisphere (*t*_(10)_ = − 2.626, *p* = 0.030) after escitalopram treatment. In Fig. 3c, during the stage-1 sleep stage, the RE of the θ band in the left hemisphere was significantly higher than that in the right hemisphere (*t*_(10)_ = − 2.626, *p* = 0.017) after escitalopram treatment. In Fig. 3f, during the REM stage, the β_2_ band in the right hemisphere showed a significant decrease compared with that in the left hemisphere (*t*_(10)_ = 2.302, *p* = 0.05) after escitalopram treatment.

Secondly, we also explored differences in non-linear LZC and C0C values between left and right hemispheres during different sleep stages. As shown in Fig. 3g, during the REM sleep stage, the LZC values did not differ between the left and right hemispheres before treatment. However, a significant decrease in the LZC value in the right hemisphere compared with that in the left (*t*_(10)_ = 4.632, *p* = 0.001) was found after escitalopram treatment.

### EEG response among different brain regions

Since escitalopram treatment may have brain region - specific effects, we investigated the EEG response in different cortical areas (frontal, central and occipital) in MDD patients (before and after treatment). EEG features of frontal cortex were average values of two channels including F3 and F4; EEG features of central cortex were average values of two channels including C3 and C4; EEG features of occipital cortex were average values of two channels including O1 and O2.

**Fig. 4:**
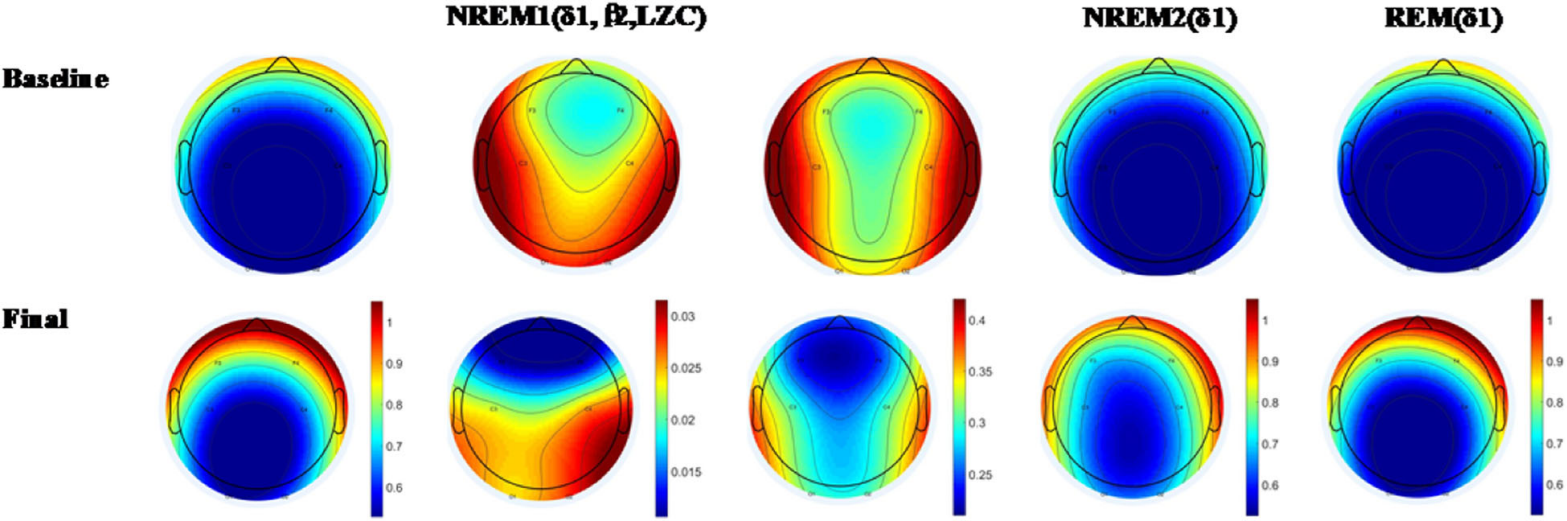
EEG topographic maps of the energy ratio and non-linear LZC values in stage-1, stage-2 and REM before and after treatment. The colors reflect the intensity of the EEG response

We found significant changes in the frontal cortex in stage-1, stage-2 and REM sleep stage after treatment. As shown in Fig. 4, for stage-1, we found that the δ_1_ band RE significantly greater (*F*_(2,30)_ = 6.961, *p* = 0.003) in the frontal cortex compared with that in the central and occipital cortices, which illustrated by warmer colors in the frontal lobe. In the same sleep stage, the β_2_ band RE (*F*_(2,30)_ = 3.928, *p* = 0.031) and the non-linear LZC values (*F*_(2,30)_ = 6.176, *p* = 0.006) in the frontal cortex significantly smaller compared with that in the central and occipital cortices, which illustrated by colder colors in the frontal cortex.

For the stage-2 and REM sleep stages, the δ_1_ band RE significantly greater in the frontal cortex, (stage-2: *F*_(2,30)_ = 6.863, *p* = 0.004); REM: (*F*_(2,30)_ = 5.740, *p* = 0.008), which illustrated by warmer colors in the frontal lobe. These results showed a more intense EEG response in the frontal cortex than that in any other brain regions.

## Discussion

With regard to temporal dynamics study, power spectrum results showed that after treatment, the δ_1_ band RE significant increased, whereas the β_2_ band RE significantly decrease. Previous studies have found that the δ_1_ band was seen during phases of reduced alertness and sleep [28]. The β_2_ frequency range is thought to reflect behavioral arousal and attention processes [21, 37]. Therefore, an increase in the δ_1_ band and a decrease in the β_2_ band may be consistent with an improved sleep quality.

It is notable that after escitalopram treatment, non-linear LZC and C0C values also showed a significant overall decrease in different sleep stages and throughout the night sleep. A decrease in LZC and C0C values may correspond to a decrease in brain wave activity and an increase in lethargy. Therefore, the non-linear dynamic features also revealed that escitalopram can improve sleep quality in MDD patients. Until now, there have been few reports looking at changes in non-linear LZC and C0C values before and after escitalopram treatment, especially during the sleep process.

The right and left side of the brain are asymmetric in anatomy and function. A previous review electrophysiological (EEG and event-related potential), behavioral (dichotic and visual perceptual asymmetry), and neuroimaging (PET, MRI, NIRS) evidence of right-left asymmetry in depressive disorders [10]. Our spatial dynamic study also found that after escitalopram treatment, sleep EEG responses in the left and right hemispheres were asymmetrical. For the stage-1, the RE of θ band in the left hemisphere was higher than that in the right. Given the association between θ band activity and rostral anterior cingulate cortex activity, an asymmetry in θ activity may reflect rapid escitalopram - induced activity within the default mode network. This in turn may indicate continued re-establishment of fronto - cingulate connections, which may be required to relieve depressive symptoms.

For the stage-2, the RE of δ_1_ band in the right hemisphere was significant higher than that in the left after escitalopram treatment. This result is in line with that of Baskaran’s research looking at power spectrum changes under closed eye conditions. In which Baskaran et al. found that escitalopram responders showed greater delta power in the right hemisphere at 2-week of escitalopram treatment [8]. Additionally, right lateralization of delta in escitalopram responders are similar to reports of increased slow wave activity in the right hemisphere in MDD patients [23]. Therefore, this feature may reflect a subtype of MDD patients that respond well to escitalopram.

For the REM sleep stage, the RE of β_2_ band was lower in the right hemisphere than that in the left after escitalopram treatment. Previous research has found that the EEG beta power has been shown to have a temporal association with cortisol secretion suggesting a mechanistic link between increased hypothalamic - pituitary - adrenal function and higher frequency brain activation [11]. In addition, changes in beta asymmetry observed in the patients after escitalopram treatment may reflect antidepressant induced variations in arousal. Differences in LZC values between the right and left hemispheres in reaction to escitalopram treatment have not yet been explored. In our study, the LZC values of the right hemisphere were lower than that in the left hemisphere during the REM sleep stage after escitalopram treatment. The neurobiological basis of this finding in the context of response to escitalopram treatment is poorly understood, and this finding needs to be further explored and verified in a wider range of studies.

Spatial dynamic research of brain region-specific targets demonstrates that after escitalopram treatment, the frontal cortex showed a more intense EEG response compared with the central, and occipital cortices. Previous research has found that the frontal lobe has a regulatory role in emotional cognition [19]. The prefrontal cortex is rich in 5-HT2A receptors, and pharmacological studies have shown that 5-HT2A receptors are involved in antidepressant behaviors [4], and that they may play an antidepressant role by increasing the release of 5-HT. Therefore, the greater the response of the frontal cortex, may be indicative of a good response to escitalopram treatment in MDD patients.

In summary, the spatio-temporal dynamics of the EEG features during sleep in MDD patients with escitalopram treatment was explored in this study. Our findings may aid in unravelling the mechanisms underlying the action of escitalopram treatment. However, these results were based on a small sample size, and therefore, larger sample size will be needed to verify them for future studies.

## Conclusions

The findings presented within this study are encouraging in several aspects. Firstly, temporal dynamics study demonstrated that there appeared an increase in the δ_1_ band, a decrease in the β_2_ band, and a decrease in non-linear LZC and C0C values after escitalopram treatment. Secondly, spatial dynamic study indicated that sleep EEG responses in the left and right hemispheres were asymmetrical, the frontal cortex showed a more intense EEG response compared with the central, and occipital cortices. These findings may contribute to a comprehensive understanding of the pharmacological mechanism of escitalopram in the treatment of depression.

## Data Availability

All datas are included in this manscript.
